# Foliar Application of Salicylic Acid at Different Phenological Stages of Peach Fruit CV. ‘Flordaking’ Improves Harvest Quality and Reduces Chilling Injury during Low Temperature Storage

**DOI:** 10.3390/plants10101981

**Published:** 2021-09-22

**Authors:** Irfan Ali, Xiukang Wang, Mohammad Javed Tareen, Fahad Masoud Wattoo, Abdul Qayyum, Mahmood Ul Hassan, Muhammad Shafique, Mehwish Liaquat, Sana Asghar, Tanveer Hussain, Sajid Fiaz, Waseem Ahmed

**Affiliations:** 1 Department of Horticulture, PMAS-Arid Agriculture University Rawalpindi, Rawalpindi 46300, Pakistan; ch.tanver@gmail.com; 2 College of Life Sciences, Yan’an University, Yan’an 716000, China; 3 Agriculture Research Institute, Quetta 87300, Pakistan; jdtn69@yahoo.com; 4 Department of Plant Breeding & Genetics, PMAS-Arid Agriculture University Rawalpindi, Rawalpindi 46300, Pakistan; fahad.pbg@uaar.edu.pk (F.M.W.); mhassan@uaar.edu.pk (M.U.H.); 5 Department of Agronomy, The University of Haripur, Haripur 22620, Pakistan; aqayyum@uoh.edu.pk; 6 Department of Horticulture, Sub-Campus Burewala, University of Agriculture Faisalabad, Vehari 61010, Pakistan; shafiqhort@hotmail.com; 7 Institute of Hydroponic Agriculture, PMAS-Arid Agriculture University Rawalpindi, Rawalpindi 46300, Pakistan; mehwish2454@yahoo.com; 8 Horticulture Section, Barani Agricultural Research Institute, Chakwal 48800, Pakistan; sanaasghar14@yahoo.com; 9 Department of Plant Breeding & Genetics, The University of Haripur, Haripur 22620, Pakistan; sfiaz@uoh.edu.pk; 10 Department of Horticulture, The University of Haripur, Haripur 22620, Pakistan; dr.waseemahmed@uoh.edu.pk

**Keywords:** peach, SA, pre-harvest, post-harvest

## Abstract

Peaches are well-liked amongst the stone fruits in Pakistan. The peach industry faces significant losses, from harvesting to marketing. The objective of this study was to investigate the effectiveness of foliar sprays of salicylic acid (SA) on the fruit quality of peaches (cv. ‘Flordaking’) at the harvest and postharvest life or stages. Different concentrations of SA (control, 1, 2 and 3 mM) were sprayed on the plants at three growth stages of fruit, i.e., the cell division, cell enlargement and pit-hardening stages. In general, all the SA treatments improved the fruit quality at harvest and maintained higher levels of flesh firmness, titratable acidity and ascorbic acid during storage. However, fruit weight loss, soluble solid contents, membrane leakage, chilling injury, color development, disease and decay incidence and the climacteric peak of ethylene were lowered by SA treatment after six weeks of low-temperature storage. SA at a 3-mM concentration was proven to be the most effective in maintaining the quality for a longer period of time during low-temperature storage. Based on the results, it can be concluded that the application of SA at fruit development stages can improve the harvest quality and storability of ‘Flordaking’ peaches.

## 1. Introduction

Peaches and nectarines are known to lose their quality rapidly after harvest, especially when stored at ambient conditions [1]. The cold storage of harvested peaches is indispensable to reduce the quality loss and excessive softening and to prolong the time for marketing by lowering the incidence of decay [2]. The storage of commodities at a low temperature is a common practice to slow down the metabolic activities of fruits [3]. Low-temperature storage not only slows down the ripening but also protects the fruit from disease and rotting incidences. Contrarily, peaches face the problem of chilling injuries when stored at a low temperature for an extended period [2]. Peaches, when stored at a low temperature undergo important physiological, textural and biochemical changes, which are mainly related with alterations in the composition and structure of the cell wall due to enzymatic action in the pectic molecules [2]. The symptoms of chilling injuries include flesh browning, flesh mealiness and woolliness [4].These symptoms normally appear when peaches are kept at room temperature following extended low-temperature storage. Chilling injuries result in mealiness, red or brown discoloration of fleshy tissues, a loss of flavor and the fruit failing to ripen uniformly [4]. Flesh browning is considered as a main chilling injury (CI) symptom caused by the oxidation of phenolics, which leads to the discoloration and rotting of fruits, limiting the storage life and causing appreciable economic losses [4]. 

Decay caused by pathogens is an important factor that limits the storage life of peaches after harvest and results in appreciable losses at the wholesaler, retailer and consumer levels [3]. The control of post-harvest diseases of fruits and vegetables mostly depends on a controlled environment, refrigeration and fungicides [5]. Of these, fungicides are the most effective tools to reduce post-harvest decay and extend the shelf life of the produce. However, fungicides are becoming less effective because of the development of pathogen resistance, and also, there are consumer concerns about possible human health risks associated with the use of fungicides [6]. The use of an induced resistance in harvested crops holds promise as a new technology for the control of post-harvest diseases. Both physical and biological agents can elicit resistance responses in harvested fruits and vegetables [7].

Salicylic acid (SA), a plant phenolic, plays a vital role in regulating the growth and development of plants [8]. It regulates stoma movement, sex expression, ion absorption and seed germination and stimulates the resistance against various biotic and abiotic stresses [9]. SA (SA) has been reported to reduce chilling injuries (CI) in maize plants, tomato fruits and banana seedlings [10,11,12]. SA enhances the chilling tolerance by regulating H_2_O_2_ metabolism and strengthening the antioxidant defense system [12,13]. In another study, the exogenous application of SA has been reported to reduce the spoilage percentage in peaches by controlling the electrolyte leakage (EL), decreasing ethylene production and respiration and maintaining firmness of the fruit [14]. 

The quality of the fruit can only be improved when the fruit is still attached to the plant, while it can be maintained for a reasonable duration in proper storage. To our knowledge, there is very little work done on the role of the pre-harvest use of SA in peaches during the critical developmental stages of the fruit, i.e., the cell division, pit hardening and cell enlargement stages, to improve the quality of peach fruits at harvest and during post-harvest storage at low temperatures. Therefore, the present study is designed to probe the role of the foliar application of SAs to improve the quality and storability of peach fruit cv. ‘Flordaking’ at a low temperature.

## 2. Results

### 2.1. Fruit Quality Attributes at Harvest

#### 2.1.1. Effect of SA on Fruit Physical Characteristics at Harvest

The effect of SA on the physical characteristics of fruit at the time of harvest for the first and second years is presented in Table 1. The fruit weight, diameter and pulp-to-stone ratio increased significantly in the treated fruits. A significant increase in fruit weight was noted in both the years by all the treatments as compared to the untreated fruit, except the salicylic (SA) 1-mM treatment. Moreover, the data of both the years showed a significant increase in the fruit diameters for two treatments, i.e., T2 and T3, as compared to the control. The maximum fruit weight and diameter were obtained by spraying 3-mM SA for both years (Table 1). Furthermore, a higher pulp-to-stone ratio was recorded for T3, followed by T2.

The effect of all the treatments on the yield of ‘Flordaking’ peach trees is shown in Table 1. The data show that all the treatments effectively increased the fruit yield, except the SA 1-mM treatment in the second year of the study. The maximum fruit yield was achieved by spraying SA at 3 mM for both the seasons, while the lowest fruit yield per plant was recorded in the control as compared to rest of the treatments. 

The values of the fruit firmness also significantly increased during both the years as compared to the control (Table 2). However, the treatment at 3-mM SA positively regulated the fruit firmness, as evident by the highest firmness values for this treatment as compared to the rest of the treatments, including the control. In contrast to this, the lowest firmness in the fruits was observed in the control. The data in Table 3 show that the SA treatments affected the fruit maturity on the tree, since the control and SA 1-mM-treated fruits were harvested on the same date based on their physical appearances while higher doses of SA delayed the physiological maturity; due to which, the date of harvesting was delayed two to three days (Table 1). 

#### 2.1.2. Effect of SA on the Chemical Characteristics of the Fruits

The results of the chemical characteristics of the fruits revealed that the total soluble solid contents (TSS) and total sugars were not affected significantly by different treatments (Table 2). The results regarding the total acidity revealed that the total acidity increased significantly by the foliar application of SA as compared to the control. The lowest total acidity of fruit juice was observed in the control fruit, while the highest acidity value was recorded in the fruits treated with 3-mM SA (Table 2). 

The ascorbic acid contents of the fruits were also affected significantly by different treatments (Table 2). The effect of the 3-mM SA treatment was more apparent as compared to the rest of the treatments in increasing the ascorbic acid contents. The lowest ascorbic acid contents were noticed in the control treatment.

#### 2.1.3. Performance of Fruit during Storage

##### Fruit Weight Loss

Overall, the results showed that all the peach fruits lost weight during storage, irrespective of the treatments. However, the fruit loss weight was much more accelerated in the control treatment as compared to the SA treatments for both years under study (Table 3). A minimum weight loss was noted in the fruits treated with 3-mM SA followed by 2-mM SA (*p* < 0.05) as compared to the control for both the years. The increase in weight loss was more pronounced during the storage period of 4–6 weeks. After 6 weeks of cold storage, the minimum physiological losses in weight (PLW) (21.88 and 27.59) were observed in the peach fruits harvested from the trees treated with 3-mM SA, whereas the highest weight losses (35.15 and 34.66) were recorded in the fruits harvested from the control trees in the first and second years, respectively. In general, all SA treatments significantly reduced the rate of weight loss in the peach fruits during the 6 weeks of low-temperature storage, regardless of the concentration of SA and time of spray application.

##### Fruit Firmness

The firmness of ‘Flordaking’ peaches reduced linearly with the advancement of the storage period (Table 3), reaching the minimum levels after six weeks in the untreated peach fruits (Table 3) for both years. It is noteworthy that all the SA treatments significantly delayed the softening process of the fruits. The results showed that the application of SA on the fruits while still attached to the trees significantly maintained their firmness during storage as compared to the control fruits, which were only sprayed with distilled water. SA at a 3-mM concentration maintained higher levels of firmness, followed by 2-mM and 1-mM concentrations of SA spray. On other hand, the fruits that did not receive any treatment (control) had the minimum value of fruit firmness both at the time of harvest and at the last day in storage. The fruits treated with 3-mM SA maintained 1.68- and 1.43-fold higher firmness than the control for the first and second years, respectively.

##### Fruit Skin Lightness (L*) and Fruit Skin Color Index (a*/b*)

The measurement of skin brightness and the darkness of peach fruits for all the treatments were observed by recording the L* values. The results depicted that a significant decrease in the skin brightness was observed in the fruits during storage irrespective of the treatments (*p* < 0.05) for both experimental years. The treated peaches significantly resisted the changes in the fruit skin color as compared to the control during storage. Significantly higher levels of L* values (skin and flesh) were observed in the fruits treated with 2-mM and 3-mM SA at the end of the 6 weeks of low-temperature storage (Table 3). The peach fruit collected from the control (untreated) trees had the lowest L* values at harvest, as well as during the 6 weeks of cold storage.

In contrast to the fruit skin brightness, the fruit skin color index increased with the advancement of the storage period (Table 4), which is an index of fruit ripening. The minimum increase in the skin color index value was observed in the 3-mM SA treatment, while the maximum increase was found in the control (Table 4) during both of the experimental repetitions (first and second year). 

##### Effect of SA on TSS, TA and TSS/TA 

At the time of harvest, the data collected for TSS depicted that all the fruits had the same level of TSS irrespective of SA treatment (Table 4). However, during storage, the TSS gradually increased while the TA decreased significantly for all the treatments during storage at 1 ± 1 °C (Table 4). This antagonistic change in the TSS and TA was significantly low in SA-treated fruit as compared to the control, which exhibited a higher level of TSS (12.2 and 11.93 °Brix). Among the SA treatments, the application of 3-mM SA treatment showed the minimum TSS value (10.7 and 11.27 °Brix), followed by the 2-mM (11.3 and 11.77 °Brix) and 1-mM SA treatments (11.82 and 11.83 °Brix), respectively, for both the years. 

A gradual decrease in the TA was observed with the passage of storage time for both the years in all the treatments at a low temperature (Table 4). However, a sharp decline in the TA was observed in the control as compared to the treated fruits. At the end of storage, significantly higher values of TA were recorded in all the treatments. The treatment of 3-mM SA showed the maximum value (0.88 and 0.77%), followed by the 2-mM SA treatment (0.85 and 0.77%), in comparison with the control treatment, which showed the lowest value (0.69 and 0.63%) at the end of the storage (Table 4). 

SSC:TA, which is considered a good index for the assessment of fruit ripening, increased progressively with the storage, the increase being significantly greater in the control than in the treated fruit (Table 5). At the end of the storage, the values of the ripening index (SSC:TA) in the control fruits were 17.84 and 19.03, while, for the 3-mM SA treatment, the values were 12.21 and 14.57, respectively, for both the studied years. For all the fruits (treated or untreated), an increase in the ripening index (SSC:TA) was attributed to a decrease in the TA, with an increase in the soluble solid contents.

##### Vitamin C

During storage, different doses of the SA treatment were found to be effective in maintaining the ascorbic acid contents of peach fruits. ‘Flordaking’ peach fruits treated with 3-mM SA showed significantly higher levels of ascorbic acid (AA) as compared to the rest of the treatments during the storage period at a low temperature (Table 5). In comparison to the untreated (control) fruits, all the treatments of SA significantly increased the level of AA during six weeks of storage (Table 5). These findings suggested that SA was quite effective in slowing down the oxidation and degradation of the AA contents of the peach fruits during storage.

##### Biosynthesis of Ethylene

The treatments did not delay the climacteric rise in ethylene production in the untreated fruit, yet significantly (*p* < 0.05) lowered the climacteric peak of ethylene during post-harvest ripening at low-temperature storage (Table 5). This reduction in the ethylene peak was directly proportional to the concentration of SA applied. Perceptibly, the 3-mM SA application proved to be more efficient in reducing the rate of production of this ripening hormone in peaches. Although all the treatments significantly lowered the ethylene climacteric peak, the lowest climacteric peaks (1.6- and 2.2-fold lower than the control during the first and second years, respectively) were observed in the fruits treated with 3-mM SA.

### 2.2. Chilling Injury (Browning Index) and Electrolyte Leakage

Peach fruits are very prone to chilling injuries when they are stored at low temperatures after harvest. The results obtained clearly demonstrated that all the treatments of SA were effective at lowering the incidence of internal browning (chilling injury) of ‘Flordaking’. Fruits receiving different treatments of SA at 1 mM, 2 mM and 3 mM had significantly decreased chilling injury indices (CI) compared to that of the control fruits (Table 6) when the peach fruits were kept at room temperature for three days, subsequently, after four weeks of low-temperature storage. All the treatments of SA were statistically on par. In the present study, the lowest levels of electrolyte leakage from the peach skins were observed in the fruits collected from the trees treated with 3-mM SA, irrespective of the time of spray application for the two consecutive years of experimentation.

### 2.3. Disease and Decay Incidence

The pre-harvest foliar spray of SA was effective in reducing the incidence of disease and decay during storage (Table 6). The data showed that all the treatments significantly minimized the incidences of disease of the stored fruits compared to the control. The untreated fruits exhibited the maximum percentages (50.67% and 51.66%) of disease incidence at the end of the storage. The minimum disease incidence was recorded in the fruits that received 3-mM SA pre-harvest sprays (14.67% and 16.66%), followed by 2-mM SA (20% and 26.66%) and 1-mM SA (23% and 31.66%). All the SA treatments were statistically on par in minimizing the incidences of disease during storage.

## 3. Discussion

The obtained results indicated that SA significantly affected the quality attributes (fruit weight, diameter, SSC and TA) at harvest. Furthermore, during storage at low temperatures, SA significantly reduced the decay percentage and weight loss. Other compositional changes such as the TA, SSC, AA and ethylene biosynthesis of peaches stored at a low temperature were slowed down significantly as compared to the control. The treatments delayed the ripening process more effectively and with a minimum quality loss as compared to the control sample, which had greater compositional changes with a maximum quality loss during storage. 

### 3.1. Effect of Pre-Harvest Treatments of SA on Fruit Quality Parameters at Harvest

In the present study, higher doses of SA delayed the initial harvest dates of the fruit. SA has been reported to delay harvest maturity in dates in jujuba when applied before harvest [15]. The delaying effect of SA might be because of its inhibitory effect on ethylene biosynthesis. The foliar application of SA significantly improved the fruit physical characteristics like fruit weight, size and pulp-to-stone ratio. This, in turn, would result in a greater economic value, since large-sized fruits are more appreciated in the market. Previous studies reported that the growth elicitors were able to improve the physical characteristics of stone fruits [16,17,18]. The improvement in fruit physical properties as a result of treatments might be due to their influence in enlarging the cell size and enhancing the strength of carbohydrate sinks, thus increasing the fruit size and weight. In this connection, it has been reported that polyamines are essential for cell growth and differentiation, and their intracellular concentration increases during periods of rapid cell proliferation [19].

The results of this study indicated that a pre-harvest treatment with SA produced fruits with a firmer texture. The softening of fruits is one of the common physical parameters to assess the progress of ripening [20], and softening is a major problem of peaches that limits their quality. The key factors associating with fruit softening are the depolymerization and degradation of the cell wall components. Srivastava and Dwivedi [20] reported that polygalacturonase is primarily responsible for the ripening associated with pectin degradation and fruit softening. The level of polygalacturonase activity has been positively correlated with fruit ripening and softening in banana and tomato fruits. The application of SA is useful in inhibiting tissue softening in fruits by reducing the cell wall hydrolases activities and maintaining a cell membrane consistency [21]. Wei et al. [22] reported that the exogenous application of SA enhances the defense mechanisms and the production of antioxidants in fruits during storage, which leads to a decrease in the lipid peroxidation of the cell membrane and results in a maintained cell membrane structure. This result was in agreement with the reports of Babalar et al. [23] and Shafiee et al. [24] that suggested pre- and post-harvest applications of SA on strawberry could decrease the softening and keep them firm during storage. Zhang et al. [25] showed that SA effectively prevented kiwifruit softening during storage and the rate of fruit ripening related to the internal SA concentration. Srivastava and Dwivedi [20] reported that SA treatment inhibited the process of banana fruit softening during ripening. Srivastava and Dwivedi [20], Zhang et al. [25] and Wang et al. [8] reported that the rapid softening of fruits during ripening was simultaneous, with a rapid decrease in the endogenous SA of fruits.

The treatments had no effect on the soluble solid and total sugar contents at harvest, while the sprays of SA at different concentrations were effective in maintaining the higher level of titratable acidity and ascorbic acid. A rapid increase in the respiration rate of untreated fruits (ethylene production and fruit senescence) appeared to provoke organic acid consumption, leading to a reduction of the titratable acidity of the fruits as compared to the treated fruit. High acid contents in SA-treated fruit might be due to slowing down the fruit respiration rate by hindering ethylene production. An increased ascorbic acid content in fruits treated with a SA may be ascribed to the suppression of ascorbate oxidase activity as a result of increased levels of endogenous polyamines in fruit pulps. The effects of SA on the activities of ascorbate oxidase in peaches are yet to be investigated. 

### 3.2. Effect of Pre-Harvest Treatments of SA on Fruit Quality Parameters during Storage

#### 3.2.1. Weight Loss

The water contents of fruits and vegetables are the main factors in maintaining the quality of the horticultural product. The post-harvest life is important for long-term storage; thus, low rates of weight loss and delay in the softening of fruits and vegetables are the key desirable traits for a long shelf life [26]. The results of the present study are in accordance with the findings of Tareen et al. [3], who found that the post-harvest application of SA significantly reduced the losses in weight as compared to the control in peaches. Previous findings also revealed that the exogenous application of SA delayed the weight loss in peaches [27], strawberries [24], apples [28], tomatoes [29] and kiwifruits [30]. The reduction in the physiological weight loss due to SA is thought to be because of the inhibitory action of SA on ethylene biosynthesis [20]. Slowing down the respiration rate by stomata closure is another phenomenon of SA that is helpful in reducing the losses during storage [31].

The deformation of fruit was influenced by both the pre-harvest treatments and storage period; thus, the firmness decreased with the prolonged storage. At the end of the storage, the maximum firmness was observed in the 3-mM SA treatment and the minimum in the control. These results are in harmony with the previous findings, which reported that the application of MeSA cut the softening process and maintained firmer fruits during storage [32]. The softening that occurs in any fruit is primarily due to a change in the cell wall carbohydrate metabolism, resulting in a net decrease in certain structural components [33]. The changes in the cell wall composition result from the action of hydrolytic enzymes produced by the fruits [34]. Srivastava and Dwivedi [20] reported that polygalacturonase is primarily responsible for ripening-associated pectin degradation and fruit softening. The level of polygalacturonase activity has been positively correlated with fruit ripening and softening in banana and tomato fruits. Application of SA is useful in inhibiting tissue softening in fruits by reducing the cell wall hydrolases activities and maintaining the cell membrane consistency [21].

Zhang et al. [25] explained that the internal SA concentration retarded the rate of fruit ripening. Similarly, Wang et al. [8] also reported that fruit softening and ripening were in concurrent with a decrease in the endogenous SA concentration of the produce. Our findings also confirmed that a reduction in softness by the pre-harvest application of SA was related to the inhibition of ethylene biosynthesis, which may be mostly due to inhibition in the conversion rate of 1-amino cyclo-propane 1-carboxylic acid (ACC) to ethylene by SA [35].

#### 3.2.2. Fruit Skin Color Brightness/Darkness and Color Index

The decline of fruit skin brightness/darkness is because of the darkening of the skin and pulp that occurs due to the browning and rises in the concentration of the pigments. The results of this study showed that fruits treated with SA had higher L* values as compared to the control, which is also in agreement with the findings of Delwiche and Baumgardner [36], who reported that a water loss from the surfaces of peach fruits caused a decreased luminosity. Tareen et al. [3] reported that peach fruits, even when given post-harvest SA treatments, maintained higher L* values as compared to untreated fruits at low-temperature storage. The least values of skin brightness (L*) were recorded in the fruits that did not receive any SA treatment, which made the fruits look darker. On the other hand, a higher skin brightness (L*) values in SA-applied fruits might be due to the reduced degradation of the chlorophyll contents and lesser weight loss. Peach fruits having high L* values were reported with good visual quality [37].

The color readings of a* denote green or red color when its value is negative or positive, respectively, and color readings of b* denote blue (nonexistent for persimmon) or yellow when the reading is negative or positive, respectively. Thus, the a*/b* ratio indicates greenness when negative and redness when positive. The fruit skin color (redness) index (a*/b*), which is a good index of ripening during storage, gradually increased during six weeks of low-temperature storage. The increase was rapid and high in untreated and SA 1-mM-treated fruits, while the fruits treated with higher concentrations of SA significantly slowed the changes in the fruit skin color index throughout the six weeks of low-temperature storage. In this experiment, accelerated a*/b* values were observed for all the treatments during low-temperature storage. The SA treatments appeared to retard the fruit skin color development, as evident from the data. During the entire storage period, untreated fruits retained higher values of a*/b*, whereas the minimum values for a* and b* were recorded in SA for the 3-mM-treated fruits. The results of this study are similar to the findings of Fattahi et al. [30], who reported a reduction in the color development of kiwifruit during storage by the application of SA. The same results were obtained from pre- and post-harvest SA applications on strawberries [23], but Shafiee et al. [24] reported that the SA treatments were not effective in lowering the redness in comparison with the control. The redness (a*/b*) value is a useful index of maturation and the degree of ripening in fruits, and the external color is a key factor indicating the quality of most horticultural commodities [21]. Changes in the redness result in increased respiration rates during storage. The SA treatment causes a decrease in respiration and a delay in the appearance of the climacteric peak, as evident in the present study, which is inversely proportional to the concentration used [20]. Shafiee et al. [24] reported that the effect of SA treatments might be due to the reduction of respiration, and it prevents an increase in the a* value, so it could have an advantage in delaying the senescence. 

#### 3.2.3. Disease and Decay Incidence

Fruits receiving a pre-harvest treatment of SA showed lower levels of decay as compared to that of the fruits of the control set after 6 weeks of low-temperature storage. Although SA does not have direct fungicidal effects, different results show that it affects and decreases the fungus development [38]. Cao et al. [39] attributed the defense-relevant properties of SA because of maintaining higher defense-relevant enzymes (peroxidase, chitinase and phenylalanine ammonia-lyase). Salicylates are major components of the signal transduction pathways of plants, playing an important role in disease resistance [40]. Babalar et al. [23] reported that SA in a concentration-dependent manner from 1 to 2 mM effectively reduced fungal decay in Selva strawberry fruits. SA applied to either a plant’s vegetative stage, fruit development stage or post-harvest stage could completely control decay and increase the fruit shelf life. Yao and Tian [41] showed pre-harvest and post-harvest treatments of sweet cherry fruits with SA, which showed significantly lower disease percentages in storage at 25 °C than the control. Shafiee et al. [24] also obtained similar results for pre- and post-harvest SA treatments on strawberry fruits. Similar results were also reported earlier in fruits like peaches, tangerines and cherries [8].

### 3.3. Chemical Parameters

Titratable acidity, along with soluble solids, is frequently used as the maturity index [42]. Acidity retains the fruit flavor during ripening [43]. Citric, quinic and malic acids are the major organic acids found in different horticultural produces. These significantly affect the taste of fruits. The conversion of these organic acids into sugars with the advancement of the ripening affects the taste and quality badly, as a good blend of SSC:TA is essential for a desirable taste and aroma. A decrease in the acidity is associated with the onset of ripening and senescence. After harvest, due to metabolic activity, these organic acids are utilized as substrates for respiration by the fruits; hence, the titratable acidity decreases [44]. Malic acid mortifies first, followed by citric acid, eventually diminishing the titratable acidity [45,46]. Soluble solids and sugars increase but sourness decreases with the onset of ripening [44]. Slower changes in the soluble solid contents (SSC) and titratable (TA) in SA (SA)-treated peaches might be mediated through the reduction in ethylene biosynthesis—hence, delayed ripening as observed for SA-treated peaches. Similar effects of SA on soluble solids and titratable acidity were noted on kiwifruit [30,47], apples [28] and tomatoes [29]. 

### 3.4. Ascorbic Acid

The decrease in ascorbic acid contents during the storage indicates the start of senesce of the fruit [48]. It is renowned that fruits are the exceptional sources of ascorbic acid. Ascorbic acid reduced significantly with the passage of the storage interval (*p* < 0.05), in all the treatments. The decrease in ascorbic acid contents during ripening is also reported in mango and apple [23,49]. Renhua et al. [50] stated that exogenous application of SA was effective in controlling ethylene production and respiration, yielding higher AA contents. Furthermore, Lam et al. [51] reported SA as anti-transparent, which protect the moisture losses and hence fruit skin browning. Moreover, exogenous applied SA could also enhance the antioxidant defense system, hence maintaining the nutritional status of vegetables and fruits that can withstand oxidation injuries better as compared to the untreated [50]. Shahkoomahally and Ramezanian [52] reported that the utilization of ascorbic acid during later storage periods may be the reason for its decreased amounts. Generally, when fruits become overripe, vitamin C content declines concurrently with the degradation of fruit tissues. The results obtained from this study indicate that the SA treatments were beneficial in delaying degradation of ascorbic acid content during storage. Therefore, The SA treated fruits exhibited higher maintenance of ascorbic acid as compared to that of control set. SA prevents vitamin C destruction by increasing the antioxidant ability and resistant of plants and fruits [8,24]. Also, exogenous SA could be effective in reducing the rate of respiration and ethylene production [50]. In the study under discussion, it was noted that SA (SA) treatment enhanced the AA contents of the fruit pulp during the storage at low temperature. In these treatments slow changes in ascorbic acid (AA) contents may be related to lower activity of the enzymes which are involved in the oxidation of ascorbic acid. Tsay et al. [53] described that continuation of respiration in kiwifruits lead to increase in ethylene content and decrease in ascorbic acid content. Earlier Bal and Celik [47] reported that the application of SA was helpful in maintaining higher amount of ascorbic acid in fruit after the storage. The results of this study can also be confirmed by previous reports of postharvest treatment with SA to preserve vitamin C contents in tomato [29], rambutan fruit [21] and pineapple fruit [54].

### 3.5. Ethylene Biosynthesis

Present results are in accordance with the findings of Babalar et al. [23], who noted significant reduction in ethylene production in ‘Selva’ strawberry by the exogenous application of SA. Zhang et al. [25] reported that postharvest treatment of kiwifruit with acetyl SA (ASA, a synthetic analogue of SA) results in lower ACC oxidase and ACC synthase activity and decreases ethylene production during the early stages of fruit ripening. Leslie and Romani [55] reported that SA contained production of ethylene in suspension- cultured pear cells. This phenomenon was considered to be as a result of its slowing up the ACC conversion into ethylene. Production of ethylene from peel flesh and seeds of apple was also significantly concealed after treatment with SA [56]. SA has also been reported in reducing both injury provoked transcription and activity of 1-aminocyclo propane 1-carboxylic acid synthase [35]. Our findings on peach fruit have corroborated these reports, including those of Xu et al. [57] describing inhibition of ACC oxidase activity in kiwifruit, and similar results in apple fruit with ASA treatments [56]. 

### 3.6. Chilling Injury Index

Peach fruits are susceptible to chilling injury when they are stored at low temperatures after harvest. In the experiment, it is clear that all the treatments of SA were effective in lowering the incidence of internal browning (chilling injury) of ‘Flordaking’ peach. Ding et al. [11] stated that MeSA (Methyl salicylate) provided protection against low temperature break down in tomato fruit. Similarly lower doses of SA provided defense against chilling stress in young maize plant [10]. In present study, the increasing concentration were better in alleviating the chilling injury incidence, though the treatments of SA were statistical non-significant with each other. The relative uptake from exogenous application as well as the effective concentration of SA alleviating chilling injury could vary depending on specie to specie, variety to variety and type of tissues.

Electrolyte leakage in the peach fruit was increased significantly by low temperature stress. SA treatments suppressed the increases in membrane leakage significantly. The optimum concentration of SA was 3 mM (*p* < 0.05) amongst all the concentrations used in the present study. Results in Table 3 show that the storage period had a significant effect on membrane leakage (*p* < 0.05). The results indicate that maximum membrane leakage was observed in control, while the lowest membrane leakage was recorded in 3 mM SA treated fruit.

Oxidation of membrane lipid causes injury which allows the reaction of polyphenol oxidase (PPO) and polyphenols (oxidizable substrates) which are usually separated causing browning [58]. High SA concentration resulted in decreased internal browning (chilling injury) which is directly associated with membrane integrity and stability [59]. As Zhang et al. [25] stated that treatments of SA on kiwifruit enhanced superoxide free radical and lipoxygenase (LOX) activity and resulted in the lower climacteric rise in ethylene biosynthesis, hence, ripening, electrolyte leakage, senescence and browning incidence were delayed [25].

Generally, chilling conditions affect mainly the membranes of cells and organelles causing alterations in the fatty acid contents of phospholipids [60,61] and then the membrane damages commence a cascade of secondary events leading to distraction of cell makeup. This damage can be measured by electrolyte leakage. These results indicate a role for SA in maintaining membrane integrity, as has been reported for loquat [62] and pomegranate fruits [63]. Zhao et al. [64] reported that the electrolyte leakage (EL) intensity exactly reflects CI development phase and degree in fruit. The results showed that SA treatment effectively alleviated symptoms and severity of CI, and possibly maintained membrane integrity, owing to reduced electrolyte leakage (*p* < 0.05). Similar results have been reported by other researchers in peach [8] and pomegranate fruits by postharvest treatments [65]. 

## 4. Materials and Methods

The present study was conducted at a private farm (Khattar Sangral Fruit Farm) in Attock District (lat.33°37′ S; long.73°06′ E) Pakistan. The facilities of cold storage and laboratory of Department of Horticulture, Pir Mehr Ali Shah-Arid Agriculture University Rawalpindi (PMAS-AAUR), Rawalpindi, Pakistan were utilized for analysis. Healthy, uniform, pest and disease-free trees were selected for the experiment. A total of 12 peach trees of cv. “Flordaking” grafted on the rootstock of ‘Peshawar Local’ having eight years of age were selected for the study. The orchard had the rectangular planting systems facing North-South direction having plant to plant 6 m × 6 m and row to row 7 m × 7 m distance. All the selected trees were given standard cultural practices. The harvested fruit was stored in the cold storage of the department of Horticulture, PMAS-AAUR, at 1 ± 0.5 °C and 90% ± 5% relative humidity while all the physiological and other chemical analysis were performed in the laboratory of the same department.

Three treatments of SA (1 mM, 2 mM and 3 mM) were applied as foliar spray at three different growth stages of fruit i.e., cell division, pit hardening or lag phase and cell enlargement stage whereas distilled water was sprayed as control treatment to the peach trees. 

Each tree was used as a treatment unit in a block and the treatments were replicated three times. Uniform sized eighty fruits free from disease, disorder and pests were harvested at horticultural maturity from each replication/ tree. Fruits were brought to the laboratory for further analysis. After washing and drying of fruits at room temperature, data for different fruit quality parameters on day of harvest was noted and the remaining fruit were packed in corrugated boxes. Fruit was stored at 1 ± 1 °C and 90% RH for 6 weeks were recorded on weekly intervals

### 4.1. Fruit Quality Parameters at Harvest

Sample of 10 mature fruits were taken to determine the physical fruit quality characteristics like fruit weight (g), fruit diameter (mm), pulp-to-stone ratio and yield of fruit per plant (Kg). Fruit firmness (N) was calculated by means of a digital penetrometer model BKD020 (WEL. New Zealand) equipped with an 8-mm plunger. The fruits used to determine the fruit firmness were then cut into smaller pieces and juice was extracted for the analysis of total soluble solids, titratable acidity and ascorbic acid contents.

Total soluble solids (TSS) were measured according to AOAC [66] using hand refractometer at room temperature. Total sugars of juice were estimated by the method described by Hortwitz [67].To determine the titratable acidity, 10 mL extracted guava juice was mixed with 40-mL distilled water, and 2 to 3 drops of phenolphthalein were added in the juice. A 10-mL aliquot was taken in a titration flask and titrated against 0.1-N NaOH until permanent light pink color appears. Three consecutive readings were taken, and the percent acidity was calculated by using the following formula:

$$\%\;\mathrm{Titratable}\;\mathrm{Acidity}\;=\frac{\frac{N}{10}\mathrm{NaOH}\;\mathrm{used}\;\times \;0.0064}{\mathrm{Volume}\;\mathrm{of}\;\mathrm{sample}\;\mathrm{used}}\times 100$$

Ascorbic acid was determined according to the method described by Hans [68]. 

### 4.2. Postharvest Performance of Peach Fruit during Storage

#### 4.2.1. Fruit Physical Characteristics

Fresh weight of fruit was recorded soon after harvest. For this purpose, 9 fruits from each tree/replication were kept separately. Weight loss of fruits from different treatments during storage at 1 ± 1 °C and 90% RH was recorded on weekly basis according to the following formula:

Weight loss (%) = (*W*1 − *W*2) × 100 / *W*1

*W1* = Fresh weight of fruit (g), *W2* = Weight after interval (g)

Firmness was noted from three fruits in each treatment after removing small piece of peel with a peach peeler at two opposite sides and determining the firmness by means of a Wagner Fruit Firmness Tester, model FT-327, having an 8-mm plunger. Values were expressed in Newton (N).

Peach fruit external color was evaluated with a Chroma Meter CR-400 (Konica Minolta 7 Sensing, Inc., Tokyo, Japan). The chromaticity coordinates, L* (lightness to darkness), were recorded at opposite sides of the fruit. Two readings were taken from each fruit. Pathogenic decay was examined at the end of the storage. The fruits having lesions, surface softening and rotting were judged decayed. The results were articulated in percentage.

Ethylene was measured on the day of harvest and then on weekly intervals till the end of the experiment. Three fruits from each replicate were weighed and then used for determination of ethylene synthesis by using gas chromatograph. Ethylene synthesis was measured according to the method described by Abbasi [69]. Three fruit sample per replicate was sealed in a jar for one hour. Then, a 1-mL sample of air was withdrawn from jar and was introduced into a GC (gas chromatograph) provided with respective column. 

#### 4.2.2. Chemical Parameters

TSS was determined from the juice of the peach by a hand refractometer according to the method described in AOAC [66] at 20 °C. Titratable acidity of freshly extracted juice from each sample was also determined by the standard method of AOAC [66]. Vitamin C (Ascorbic acid) was determined according to the method described by Hans [68].

#### 4.2.3. Chilling Injury and Membrane (Electrolyte) Leakage

The degree of chilling injury was assessed according to Wang et al. [8], while the membrane/electrolyte leakage was measured as described by Wang et al. [70].

### 4.3. Statistical Analysis

The experimental design was laid out according to Randomized Complete Block Design (RCBD) with four treatments that were replicated thrice. The data was analyzed using MSTAT-C software [71], while the Duncan multiple range test (DMRT) was used to compare differences among the treatments at 95% confidence level of each variable [72].

## 5. Conclusions

The results of this paper report for the first time the pre-harvest treatments of SA on the improvements of the fruit quality at harvest and maintaining it during storage by reducing chilling injuries and ethylene production, resulting in firmer fruit. The results suggest that the pre-harvest foliar application of SA at different phonological stages has the potential for maintaining the fruit quality by reducing the chilling injuries during low-temperature storage for peach fruit cv. ‘Flordaking’. The results also indicated that pre-harvest treatments of this chemical were equally effective in improving the quality of peaches at harvest. However, further research is needed to reveal the different mechanisms of action by which SA enhances the storage life and reduces the softening, chilling injuries and ethylene production in peach fruits.

## Figures and Tables

**Table 1:** Effects of different pre-harvest treatments of SA on the peach fruit physical characteristics at harvest in the 1st and 2nd years.

	Fruit Weight (g)		Diameter (mm)		Pulp:Stone		Harvesting Date		Yield (Kg)	
	1st Year	2nd Year	1st Year	2nd Year	1st Year	2nd Year	1st Year	2nd Year	1st Year	2nd Year
Control	123.33 b	106.67 b	53.78 c	54.42 b	9.29 b	8.92 c	0	0	44.75 c	41.87 c
SA 1	123.33 b	108.33 b	55.66 bc	54.58 b	9.79 b	10.45 b	0	0	47.38 b	42.76 bc
SA 2	125.56 b	112.22 a	58.66 ab	57.52 ab	11.94 a	11.08 b	+2	+1	48.07 b	45.00 b
SA 3	132.56 a	113.78 a	60.33 a	58.85 a	12.14 a	12.59 a	+2	+2	52.07 a	47.78 a
LSD	2.63	3.84	3.44	3.29	1.69	0.74			2.42	2.43

(+, days after; -, days before the control harvest date). Means within a column having same letters are statistically nonsignificant using the Least Significant Difference Test.

**Table 2:** Effects of different pre-harvest treatments of SA on the peach fruit physicochemical characteristics at harvest in the 1st and 2nd years.

	Fruit Firmness (N)		Total Soluble Solids (˚Brix)		Total Acidity (%)		Total Sugars (%)		Ascorbic Acid (mg/100 g FW)	
	1st Year	2nd Year	1st Year	2nd Year	1st Year	2nd Year	1st Year	2nd Year	1st Year	2nd Year
Control	75.33c	97.44 c	9.32 a	9.17 a	0.93 c	1.09 b	4.51 a	4.65 a	4.33 c	4.90 c
SA 1	83.00 b	100.00 bc	9.45 a	9.03 a	0.98 b	1.10 b	4.50 a	4.62 a	4.77 b	5.33 bc
SA 2	85.46 b	101.28 b	9.40 a	9.17 a	1.10 a	1.12 ab	4.57 a	4.79 a	5.00 ab	5.65 ab
SA 3	94.67 a	107.69 a	9.40 a	9.37 a	1.11 a	1.14 a	4.58 a	4.75 a	5.20 a	6.08 a
LSD	3.75	3.57	0.59	1.16	0.04	0.04	0.13	0.24	0.32	0.74

Means within a column having same letters are statistically non-significant using the Least Significant Difference Test.

**Table 3:** Effect of different pre-harvest treatments of SA on the peach fruit physicochemical characteristics during low-temperature storage.

SA mM (Concentration)	Storage Period (Weeks)	Weight Loss (%)		Firmness (N)		Fruit Skin Brightness	
		1st Year	2nd Year	1st Year	2nd Year	1st Year	2nd Year
0	0	0.00 L	0.00 L	75.33 C	97.43 AB	68.45 A	72.47 AB
1	0	0.00 L	0.00 L	83.00 B	100.00 AB	69.66 A	73.97 A
2	0	0.00 L	0.00 L	85.47 B	101.30 AB	69.52 A	73.67 AB
3	0	0.00 L	0.00 L	94.67 A	107.70 A	69.03 A	73.30 AB
		0.00 G	0.00 F	84.62 A	101.61 A	69.16 A	73.35 A
0	1	8.52 JK	6.09 I–L	36.35 GH	75.67 C	61.04 A–E	70.58 AB
1	1	7.33 K	5.24 I–L	41.20 FG	82.67 BC	63.30 A–D	70.89 AB
2	1	6.48 K	4.43 KL	49.60 DE	83.33 BC	69.13 A	72.76 AB
3	1	6.33 K	4.45 J–L	55.95 D	84.00 BC	69.23 A	72.99 AB
		7.17 F	5.05 E	45.78 B	81.42 B	65.68 AB	71.81 A
0	2	13.27 GHI	17.06 E–H	31.94 HI	45.67 D–G	57.79 B–E	70.16 AB
1	2	11.75 HI	13.38 G–J	45.44 EF	50.00 DE	58.84 B–E	69.84 AB
2	2	10.94 IJ	13.62 GHI	51.64 DE	53.33 DEF	65.71 AB	71.85 AB
3	2	10.71 IJ	10.95 H–K	53.53 D	54.00 D	69.04 A	71.93 AB
		11.67 E	13.75 D	45.64 B	50.75 C	62.85 BC	70.95 AB
0	3	18.74 D	24.78 B–F	18.43 JK	30.33 FGH	54.80 CDE	69.84 AB
1	3	15.92 EFG	20.13 C–G	26.75 I	34.67 D–H	58.52 B–E	68.90 AB
2	3	14.09 GH	18.72 D–H	28.25 I	35.33 D–H	63.78 A–D	70.57 AB
3	3	13.42 GHI	16.01 FGH	30.70 HI	39.67 D–H	66.01 AB	70.95 AB
		15.54 D	19.91 C	26.03 C	35.00 D	60.78 CD	70.06 ABC
0	4	23.2 C	28.59 ABC	13.39 JKL	27.00 GH	54.79 CDE	64.86 B
1	4	18.56 DE	23.84 B–F	19.16 J	31.67 FGH	58.60 B–E	68.02 AB
2	4	15.58 FG	20.83 B–G	19.16 J	34.67 D–H	64.11 ABC	67.69 AB
3	4	14.62 G	19.01 D–H	26.03 I	33.33 D–H	66.27 AB	70.36 AB
		18.04 C	23.07 BC	19.44 D	31.67 D	60.94 CD	67.73 BCD
0	5	29.94 B	29.72 AB	9.70 L	25.67 GH	53.03 E	64.83 B
1	5	21.94 C	25.43 B–E	12.97 JKL	31.00 FGH	57.04 B–E	65.61 AB
2	5	18.90 D	24.91 B–F	15.23 JKL	32.00 E–H	62.65 A–D	67.11 AB
3	5	18.25 DEF	23.45 B–F	18.23 JK	34.00 D–H	64.85 AB	68.84 AB
		22.26 B	25.88 AB	14.03 E	30.67 D	59.39 CD	66.60 CD
0	6	35.15 A	34.66 A	8.73 L	22.00 H	51.83 E	64.83 B
1	6	27.49 B	26.08 A–D	12.47 KL	28.33 GH	54.55 DE	65.67 AB
2	6	23.36 C	29.00 ABC	12.97 JKL	29.67 FGH	60.84 A–E	65.43 AB
3	6	21.88 C	27.59 A–D	14.73 KL	31.67 FGH	62.64 A–D	66.30 AB
		26.97 A	29.33 A	12.22 E	27.92 D	57.46 D	65.56 D
LSD (*p* <0.05)		2.76	8.9382	6.55	21.519	3.71	
Concentration			**		**		*
Storage period			**		**		**
Concentration × storage period			NS		NS		NS

Means within a column having same letters are statistically non-significant using the Least Significant Difference Test. ** = Highly significant, * = Significant.

**Table 4:** Effect of different pre-harvest treatments of SA on the peach fruit physicochemical characteristics during low-temperature storage.

SA mM (Concentration)	Storage Period (Weeks)	Fruit Skin Color Index (a*b*)		TSS (°Brix)		TA (%)	
		1st Year	2nd Year	1st Year	2nd Year	1st Year	2nd Year
0	0	0.17 I	0.17 IJ	9.32 J	9.17 GH	0.93 D–I	1.07 ABC
1	0	0.15 I	0.13 J	9.45 IJ	9.03 H	0.98 C–F	1.10 AB
2	0	0.15 I	0.17 IJ	9.40 IJ	9.17 GH	1.10 A	1.13 A
3	0	0.15 I	0.17 IJ	9.40 IJ	9.37 FGH	1.11 A	1.13 A
		0.15 E	0.16 E	9.39 F	9.18 E	1.03 A	1.11 A
0	1	0.35 D–H	0.35 D–H	10.52 D–G	10.33 C–F	0.88 I–L	0.96 A–F
1	1	0.31 GH	0.31 E–I	10.17 E–J	9.77 E–H	0.93 E–I	1.03 A–D
2	1	0.22 I	0.22 G–J	9.58 HIJ	9.33 FGH	1.00 CD	1.00 A–E
3	1	0.20 I	0.20 HIJ	9.50 HIJ	9.13 H	1.09 AB	1.08 AB
		0.27 D	0.27 D	9.94 E	9.64 D	0.97 B	1.02 B
0	2	0.40 DE	0.40 C–F	10.65 D–G	10.43 CDE	0.79 MN	0.97 A–E
1	2	0.38 D–G	0.38 C–G	10.32 E–I	10.47 CDE	0.87 I–L	0.95 B–F
2	2	0.32 F–H	0.32 E–I	9.99 E–J	10.20 D–G	0.96 C–G	1.01 A–E
3	2	0.30 H	0.30 F–I	9.76 G–J	9.90 E–H	1.02 BC	1.02 A–D
		0.35 C	0.35 C	10.18 DE	10.25 C	0.91 C	0.99 B
0	3	0.41 DE	0.41 C–F	11.29 BCD	11.17 A–D	0.76 NO	0.93 B–G
1	3	0.39 DEF	0.39 C–F	10.68 D–G	10.97 A–D	0.87 I–L	0.93 B–G
2	3	0.35 E–H	0.35 D–H	10.10 E–J	10.97 A–D	0.92 F–I	1.01 A–E
3	3	0.31 GH	0.31 F–I	9.96 F–J	10.50 CDE	0.98 C–F	0.99 A–E
		0.36 C	0.36 C	10.51 CD	10.90 B	0.88 CD	0.97 B
0	4	0.42 CDE	0.42 C–F	11.28 BCD	11.37 ABC	0.71 OP	0.84 E–I
1	4	0.42 CD	0.42 C–F	10.91 CDE	10.98 A–D	0.83 KLM	0.79 F–J
2	4	0.36 D–H	0.36 D–H	10.42 D–H	10.97 A–D	0.90 G–J	0.86 D–I
3	4	0.32 FGH	0.32 E–I	10.09 E–J	10.77 B–E	0.99 CDE	0.90 C–H
		0.38 C	0.38 C	10.68 BC	11.02 B	0.86 DE	0.85 C
0	5	0.54 B	0.54 ABC	11.90 AB	12.00 A	0.70 OP	0.75 HIJ
1	5	0.48 BC	0.48 A–D	11.29 BCD	11.00 A–D	0.81 LMN	0.76 HIJ
2	5	0.38 DEFG	0.38 C–G	10.57 D–G	11.17 A–D	0.89 H–K	0.79 F–J
3	5	0.40 DE	0.40 C–F	10.24 E–J	11.00 A–D	0.96 C–H	0.79 F–J
		0.45 B	0.45 B	11.00 B	11.29 AB	0.84 E	0.77 D
0	6	0.61 A	0.60 AB	12.26 A	11.93 A	0.69 P	0.63 J
1	6	0.64 A	0.63 A	11.82 ABC	11.83 A	0.75 NOP	0.70 IJ
2	6	0.49 B	0.50 A–D	11.33 ABCD	11.77 AB	0.85 J–M	0.77 G–J
3	6	0.48 BC	0.47 B–E	10.76 DEF	11.27 ABC	0.88 IJK	0.77 G–J
		0.56 A	0.55 A	11.54 A	11.70 A	0.79 F	0.72 D
LSD (*p* <0.05)		0.07	0.16	0.94	1.06	0.07	0.18
Concentration			**		**		**
Storage period			**		**		**
Concentration × storage period			NS		*		NS

Means within a column having same letters are statistically non-significant using the Least Significant Difference Test. ** = Highly significant, * = Significant.

**Table 5:** Effects of different pre-harvest treatments of SA on the peach fruit physicochemical characteristics during low-temperature storage.

SA mM (Concentration)	Storage Period (Weeks)	TSS:TA		AA (mg/100 g FW)		Ethylene Biosynthesis		Electrolyte Leakage (%)	
		1st Year	2nd Year	1st Year	2nd Year	1st Year	2nd Year	1st Year	2nd Year
0	0	9.99 I–L	8.47 IJ	4.33 B–F	4.93 BCD	9.60 N	7.33 N	44.06 H–M	35.7 E–I
1	0	9.65 JKL	8.30 J	4.77 AB	5.37 A–D	8.87 N	7.50 N	41.81 I–M	34.90 GHI
2	0	8.61 KL	8.17 J	5.00 AB	5.67 ABC	8.90 N	6.93 N	38.75 LM	35.30 F–I
3	0	8.48 L	8.13 J	5.20 A	6.13 A	8.27 N	6.83 N	36.39 M	29.50 I
		9.18 F	8.27 F	4.83 A	5.53 A	8.91 E	7.15 E	40.25 E	33.87 D
0	1	12.03 E–I	10.83 F–J	3.93 E–H	4.65 B–F	25.77 HIJ	26.59 G–J	49.53 E–J	36.61 D–I
1	1	11.00 G–J	9.51 HIJ	4.03 D–G	4.78 B–E	23.81 I–L	24.60 IJK	45.60 G–L	35.22 F–I
2	1	9.58 JKL	9.78 G–J	4.73 ABC	4.65 B–F	22.37 J–M	23.12 K	40.76 J–M	34.89 GHI
3	1	8.74 KL	8.66 IJ	4.93 AB	5.70 AB	19.56 M	18.15 M	39.21 KLM	33.70 HI
		10.34 E	9.69 E	4.41 B	4.95 B	22.88 D	23.12 D	43.78 D	35.11 D
0	2	13.57 C–F	10.73 F–J	3.63 G–J	4.38 D–I	30.17 DEF	30.39 DEF	57.45 B–E	46.33 A–H
1	2	11.84 E–I	10.06 G–J	3.70 F–I	4.30 D–I	26.64 GHI	29.21 EFG	45.08 H–M	39.06 C–I
2	2	10.40 G–L	9.81 G–J	4.57 A–E	4.55 B–H	24.35 IJK	24.45 JK	39.87 KLM	38.29 C–I
3	2	9.53 JKL	10.76 F–J	4.70 A–D	4.59 B–G	20.42 LM	20.14 LM	39.80 KLM	38.77 C–I
		11.34 D	10.34 DE	4.15 BC	4.45 C	25.39 C	26.05 C	45.55 CD	40.61 C
0	3	14.97 BCD	12.48 D–H	3.07 IJK	4.28 D–I	31.95 CD	44.00 A	59.54 A–D	48.66 A–F
1	3	12.37 EFG	11.74 E–I	3.53 G–J	4.25 D–I	31.40 CDE	37.80 B	47.35 F–L	43.01 A–H
2	3	10.98 G–J	10.68 F–J	4.37 B–F	4.53 C–H	28.20 E–H	32.73 CD	44.07 HM	41.76 B–I
3	3	10.16 H–L	10.68 F–J	4.57 A–E	4.58 B–G	22.11 KLM	22.70 KL	41.94 IM	39.94 C–I
		12.12 CD	11.40 D	3.88 CD	4.41 C	28.41 B	34.31 A	48.23 C	43.34 BC
0	4	16.05 AB	13.48 C–F	2.97 JK	3.35 IJK	39.07 A	39.36 B	62.08 ABC	51.19 ABC
1	4	13.22 DEF	13.96 B–F	3.50 G–J	3.75 E–J	33.73 BC	33.58 C	54.19 C–G	45.60 A–H
2	4	11.64 F–J	12.88 C–G	3.80 FGH	4.25 D–I	31.35 CDE	31.78 CDE	48.70 E–J	47.00 A–H
3	4	10.27 G–L	12.02 D–H	4.07 C–G	4.29 D–I	27.32 F–I	28.11 FG	47.67 F–K	44.41 A–H
		12.79 BC	13.08 C	3.58 DE	3.91 D	32.87 A	33.21 A	53.16 B	47.05 AB
0	5	17.18 A	16.07 ABC	3.03 IJK	2.90 JK	36.77 AB	39.26 B	64.42 AB	50.02 A–D
1	5	13.95 B–E	15.01 B–E	3.27 H–K	3.00 JK	30.07 D–G	31.03 CDE	54.54 C–F	48.00 A–G
2	5	11.89 E–I	14.22 B–E	3.70 F–I	3.45 G–K	26.21 HI	27.41 GHI	51.76 D–H	45.92 A–H
3	5	10.73 G–K	13.87 B–F	3.87 FGH	3.60 F–J	21.64 KLM	23.21 K	50.51 E–I	44.17 A–H
		13.44 B	14.79 B	3.47 EF	3.24 E	28.67 B	30.22 B	55.31 AB	47.03 B
0	6	17.84 A	19.03 A	2.70 K	2.33 K	33.07 CD	31.03 CDE	66.43 A	50.63 ABC
1	6	15.74 ABC	17.00 AB	3.00 JK	3.00 JK	26.47 HI	27.50 GH	61.41 ABC	55.83 A
2	6	13.28 DEF	15.23 BCD	3.57 G–J	3.03 JK	24.03 IJK	25.00 H–K	56.03 B–F	53.63 AB
3	6	12.21 E–H	14.57 B–E	3.60 G–J	3.40 H–K	21.00 KLM	24.00 JK	50.79 D–H	49.17 A–E
		14.77 A	16.46 A	3.22 F	2.94 E	26.14 C	26.88 C	58.66 A	52.32 A
LSD (*p* <0.05)		2.19	3.32	0.67	1.15	3.52	2.88	8.7740	3.45
Concentration			**		**	**	**		**
Storage period			**		**	**	**		**
Concentration × storage period			NS		NS	**	**		NS

Means within a column having same letters are statistically non-significant using the Least Significant Difference Test. ** = Highly significant, * = Significant.

**Table 6:** Effects of different pre-harvest treatments of SA on the chilling injuries and disease and decay incidences of peach fruits during low-temperature storage.

SA mM (Concentration)	Chilling Injury		Disease and Decay (%)	
	1st Year	2nd Year	1st Year	2nd Year
Control	0.2302 A	0.4500 A	50.67 A	51.667 A
SA 1 mM	0.1190 B	0.3917 A	22.67 B	31.667 B
SA 2 mM	0.1111 B	0.1583 B	20.00 B	26.667 BC
SA 3 mM	0.0873 B	0.0917 C	14.67 B	16.667 C
LSD (*p* < 0.05)	0.0645	0.0633	8.64	13.603

Means within a column having same letters are statistically non-significant using the Least Significant Difference Test.

## Data Availability

The data presented in this study are available upon fair request from the corresponding author.
